# High-Specific Power Flexible Photovoltaics from Large-Area MoS_2_ for Space Applications

**DOI:** 10.1021/acsaem.4c01797

**Published:** 2025-01-02

**Authors:** Timothy Ismael, Muhammad Aamir Abbas, Owen P. Harris, George B. Ingrish, Meghan E. Bush, Joshua M. Sasson, Jeremiah S. McNatt, Matthew David Escarra

**Affiliations:** † Department of Physics and Engineering Physics, Tulane University, New Orleans, Louisiana 70118-5636, United States; ‡ NASA Glenn Research Center, Cleveland, Ohio 44135, United States

**Keywords:** 2D transition metal dichalcogenides, flexible photovoltaics, space radiation, space solar power, high-specific power energy generation, CubeSats

## Abstract

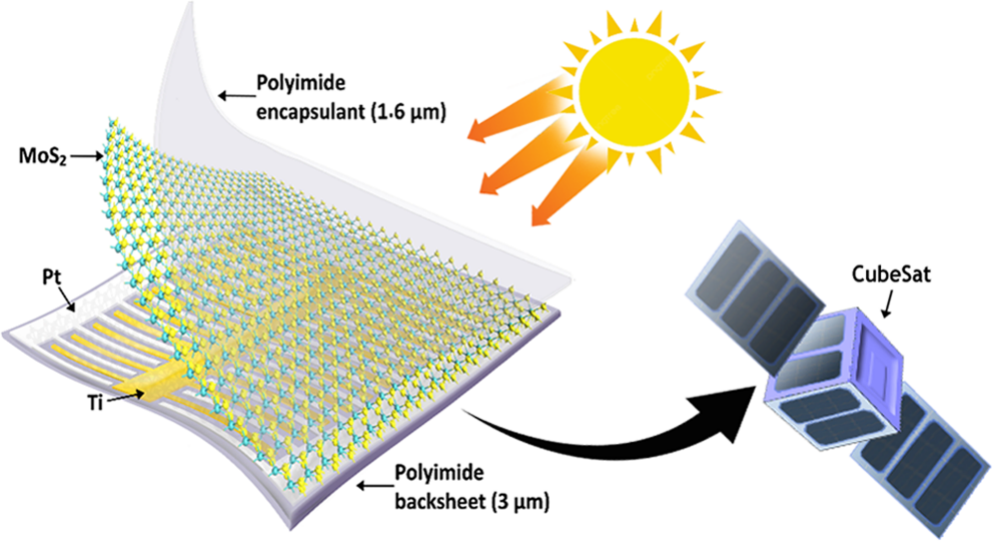

Two-dimensional (2D) transition metal dichalcogenides (TMDCs) such as MoS_2_ and WSe_2_ are excellent candidates for photovoltaic (PV) applications. Here, we present the modeling, fabrication, and characterization of large-area CVD-grown MoS_2_-based flexible PV on an off-the-shelf, 3 μm-thick flexible colorless polyimide with polyimide encapsulation designed for space structures. The devices are characterized under 1 sun AM0 illumination and show a *V*_OC_ of 0.180 V and a specific power of 0.001 kW/kg for a subnanometer-thick, single MoS_2_ monolayer absorber. Model projections indicate that the polyimide encapsulant introduces negligible absorption loss, and up to 12.97 kW/kg specific power is attainable for a 100 nm-thick MoS_2_ absorber layer. The devices maintain their performance after repetitive bending down to 5 mm bend radius. An increase in performance is measured after radiation exposure to 1 MeV e^–^ fluence, which is partially attributed to defect healing. Techno-economic analysis shows that even with a lower efficiency, the specific power of a 2D PV array designed for a 6U CubeSat is 2 orders of magnitude higher, and the cost to deploy in space is 2 orders of magnitude less than that of a Si panel used in space. This indicates that the 2D TMDC-based PV has great potential for space applications.

## 1 Introduction

Industries and researchers are continuously searching for semiconducting materials suitable for lightweight, high-specific power photovoltaics (PV). In recent years, thin-film PV technology such as cadmium telluride (CdTe), copper indium gallium diselenide (CIGS), and three-dimensional (3D) perovskites have shown some promise; however, silicon (Si) solar cells continue to be the go-to material for many applications due to the abundance of Si on earth compared to other materials, the low-cost manufacturing, scalability, and good power conversion efficiency (PCE). The high PCE requires relatively thick absorbers and is now close to its fundamental limit.^1−3^ Future space missions will depend largely on PV to power the various systems; therefore, significant research is geared toward the development of materials and systems for space-based PV. The ideal space PV requires several properties, including high PCE, an ultrathin form factor for lightweight and reduced launch costs, flexibility for ease of stowage, and resistance to radiation-induced degradation that can arise from exposure to high-energy particles in space (preserving performance and minimizing the need for weighty protective layers).^4^ However, the robustness and optical absorption of Si are reduced at ultrathin thicknesses. Furthermore, Si solar cell performance degrades significantly when exposed to high-energy protons and electrons,^5,6^ which reduces power system lifetime in missions where high radiation exposure is expected.

Thin-film PV has progressed over the years as a potential solution for space PV with good scalability. Recently, thin-film solar cells with efficiencies >20% at absorber thicknesses less than 5 μm have been made using conventional semiconductors such as III–V materials and CdTe.^7−9^ However, the cell fabrication cost has not been significantly reduced compared to Si cells and would require further reduction in thickness to compete with Si prices. Furthermore, like Si, additional thinning is accompanied by reduced absorption, mechanical strength, and efficiency, unless absorption enhancements, buffer layers to suppress recombination, and structural support are added back,^8,9^ all of which increase the mass and/or cost of the deployed module. Another emerging thin-film photovoltaic technology, perovskite solar modules, can attain PCEs up to 21.1%, with the lowest reported absorber layer film thicknesses in the 100 nm to 1 μm range and potential for high power density in the module form. However, their considerable degradation risk, due to environmental conditions such as high humidity, temperature, and ultraviolet radiation exposure that promotes photocatalysis of metal oxides in the perovskites, makes them vulnerable to significant performance loss prior to space launch and requires more robust module packaging, which affects power density.^10−14^ More detailed discussion of recent advancements and future challenges for solar cells used in aerospace applications indicates the significant opportunity for improvement in this domain.^15,16^

Alongside the development of conventional semiconductors, emerging materials such as transition metal dichalcogenides (TMDCs) are being considered for thin-film PV, particularly as a suitable option for high-specific power, flexible, radiation-resistant single, and multijunction solar cells.^15−19^ Other 2D materials, such as graphene and transmission metal carbides, have also shown potential to improve the performance and stability of solar cells.^15^ In particular, 2D TMDCs are predicted to achieve solar absorption an order of magnitude higher per unit thickness than GaAs and Si in the visible spectrum and 1 to 3 orders of magnitude higher specific power than existing ultrathin solar cells.^20−22^ A 1 nm-thick MoS_2_ solar cell can exhibit an order of magnitude higher specific power than a 1 μm-thick GaAs solar cell,^23^ and at PCEs of 9.22%, <4 nm-thick MoS_2_-based solar cells are projected to have a specific power value of >100 Wg^–1^ under 1 sun (AM 1.5) illumination.^24^ TMDCs exhibit a wide range of band gaps (∼1.0–2.5 eV),^25^ and the active layers are typically submicron, which is a significant improvement over conventional semiconductors in terms of specific power and weight. Furthermore, they are free of dangling bonds at the surface, eliminating the need for lattice matching when designing heterostructures.

The flexibility of TMDCs makes them ideal for ease of PV stowage for space deployment, and their strong in-plane covalent bonds make them relatively mechanically robust for their thickness. In one study, MoS_2_ sheets were bent down to a radius of curvature of 0.34 nm before bond breaking was observed,^26^ showing promise toward easy deployment in space by furling and unfurling 2D MoS_2_-based PV.

Due to the atomically thin nature of TMDCs, they possess exceptional radiation tolerance suitable for space applications. In one study, 2D MoS_2_ FETs maintained high currents and on/off ratios while being exposed to a He^2+^ fluence of 10^15^ ions/cm^2^ or proton fluence of 1.26 × 10^16^ ions/cm^2^, corresponding to thousands of years of proton and α particle irradiation in space.^27^ WS_2_ devices were exposed to γ-radiation of 18.41 × 10^9^ cm^–2^ sr^–1^ MeV^–1^ fluence and had increased photoluminescence (PL) and carrier lifetime, attributed to the decreased defect densities,^28,29^ highlighting the radiation hardness benefit of 2D PV.

Work has been done to demonstrate large-area 2D PV and incorporate various techniques to improve PCE.^18,30−32^ Projections show that a *V*_OC_ of up to ∼1.2 V and a FF of up to ∼81% can be obtained for an MoS_2_ solar cell,^24,33^ while a *J*_SC_ of 13.4 mA/cm^2^ under 1 sun (AM 1.5) illumination has already been demonstrated.^34^ Separately, integration of very small-area high-PCE 2D PV on flexible substrates has been shown.^35^ The PCE of TMDC solar cells is generally affected by strong Fermi-level pinning at the metal contact–TMDC interface and doping limitations, which have been addressed by the insertion of an ultrathin graphene interlayer at the metal–TMDC interface, a MoO_*x*_ capping layer for doping and passivation, and an antireflective coating, all on a 5 μm, flexible polyimide substrate; this work achieved a PCE of 5.1% and a specific power of 4.4 Wg^–1^.^36^ However, the scalability of 2D PV to large-area devices incorporating flexible substrates and their bending compatibility have not yet been demonstrated and are necessary to demonstrate their promise for applications in space.

In this work, we demonstrate the design and direct fabrication of scalable, large-area CVD-grown monolayer MoS_2_-based flexible PV devices on ultrathin 3 μm polyimide films, designed for applications in space, which are subsequently encapsulated with a 1.6 μm-thick polyimide layer. This work reports on PV performance, bending compatibility, and radiation resistance of solar cells. Additionally, the projected cell performance for devices with a thicker MoS_2_ absorber while being encapsulated between the polyimide films are calculated using the transfer matrix method (TMM) to inform the best absorber thickness for higher cell efficiency and show that the flexible 2D PV can outperform space-based Si modules in specific power. We also present the design and techno-economic analysis for the implementation of this 2D PV in a solar array on a 6U CubeSat, highlighting some of the benefits of 2D PV in space applications.

## 2 Experimental Section

### 2.1 Device Design

**Figure 1:**
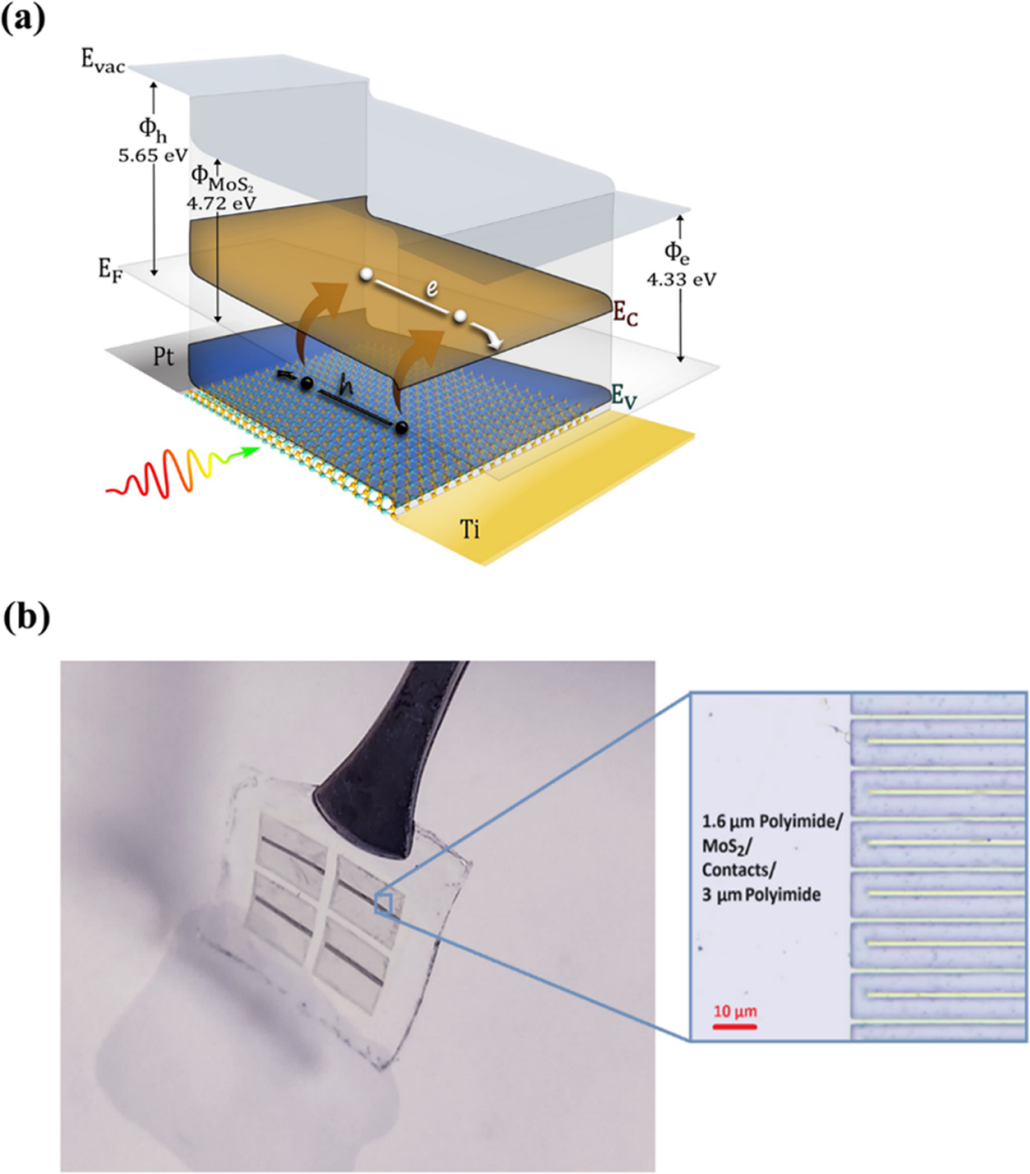
(a) Band structure of a Ti–MoS_2_–Pt Schottky-junction solar cell showing the band bending at the metal–semiconductor interfaces. (b) Optical image of large-area (25 mm^2^) ultrathin and flexible 2D PV devices.

Current cell technology such as CIGS, inverted metamorphic multijunction (IMM) III–V, and passivated emitter and rear contact (PERC) Si cells limits the active region minimum thickness to the 10–100 μm range, with a module thickness of 10 to 50 mm.^37−39^ However, ultrathin high-specific power 2D material-based solar cells can reduce active region thickness to <150 nm, with module thickness less than 10 μm, thus reducing the weight and volume per generated watt. There have been considerable efforts to demonstrate the p–n junction and Schottky-junction high-specific power thin-film PV both computationally and experimentally using single- and few-layer exfoliated 2D materials.^25,35,40−42^ Previous reports have also demonstrated large-area chemical vapor deposition (CVD) synthesis and fabrication of lateral Schottky-junction 2D MoS_2_-based PV using Ti and Pt for contact asymmetry.^18^ This work builds on this scalable approach where the high work function (Φ_h_) contact Pt is hole-selective with the metal Fermi level closely aligned with the MoS_2_ valence band, whereas the low work function (Φ_e_) contact Ti is electron-selective with the metal Fermi level closely aligned with the MoS_2_ conduction band as shown in Figure 1a.

To further optimize the aforementioned solar cell architecture for space applications, a process was developed for the direct fabrication of the cells on an off-the-shelf, strong, flexible, tear-resistant colorless polyimide (CP1) substrate produced by NeXolve for space structures,^43^ instead of the spin coating and peeling off approach commonly used to fabricate small-scale 2D material-based devices on flexible substrates. Here, the polyimide substrate was sandwiched between water-soluble poly(vinyl alcohol) layers, which served as protective layers for the polyimide from aggressive solvents during fabrication steps, such as the resist coating and liftoff steps, while providing a sacrificial layer for easy release from the handler. With a density of 1.54 g/cm^3^, a 3 μm-thick substrate and 1.6 μm encapsulant using the same polyimide add just 7.08 × 10^–4^ g/cm^2^ to the mass of the solar cell. A 1.6 μm-thick film of NeXolve’s CP1 polyimide liquid resin in diglyme with a viscosity of 2500 ± 250 cP was spin-coated onto the as-grown MoS_2_ film on its sapphire growth substrate for the transfer support film and encapsulant. The fabrication steps for the device are detailed in the Supporting Information and are shown schematically in Figure S1. By fabricating solar cells on the flexible polyimide substrate up to 10 s of mm^2^ active area, as compared to the 10 s of μm^2^ active area from exfoliated 2D TMDC-based flexible PV, we demonstrate the scalability of the flexible 2D PV device fabrication process in this work. A photograph of large-area 2D solar cells with 25 mm^2^ active area on the flexible polyimide substrate is shown in Figure 1b.

### 2.2 Device Model

**Figure 2:**
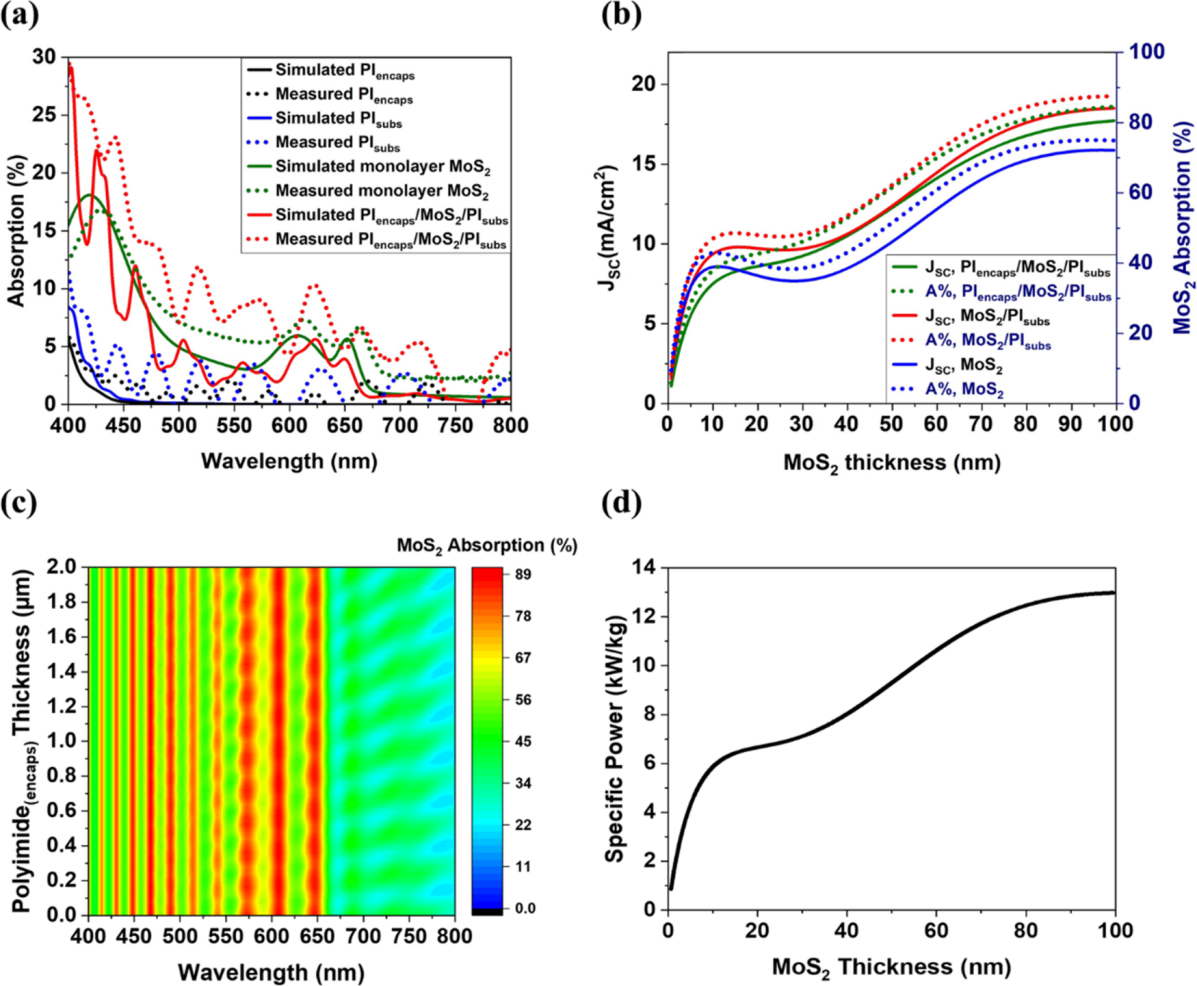
(a) Simulated and measured absorption spectra for the MoS_2_ monolayer, polyimide substrate (PI_subs_), polyimide encapsulant (PI_encaps_), and the 1.6 μm encapsulant on monolayer MoS_2_ on a 3 μm polyimide substrate stack (PI_encaps_/MoS_2_/PI_subs_). (b) Simulated *J*_SC_ and absorption vs MoS_2_ thickness using the TMM for the MoS_2_, MoS_2_/PI_subs_, and the PI_encaps_/MoS_2_/PI_subs_ stack. (c) Simulated absorption spectra in the 100 nm-thick MoS_2_ layer vs polyimide encapsulant thickness using TMM for the PI_encaps_/MoS_2_/PI_subs_ stack. (d) Simulated specific power for the 2D Ti-MoS_2_–Pt solar cell, encapsulated on a polyimide substrate, vs MoS_2_ thickness.

The absorption spectra of the polyimide layers, the MoS_2_ layer, and the MoS_2_ polyimide stack of the solar cell were measured and modeled to characterize the absorption of each layer. The absorption spectra for the MoS_2_ monolayer (0.65 nm), polyimide substrate (PI_subs_), polyimide encapsulant (PI_encaps_), and the stack after an MoS_2_ monolayer was transferred onto the polyimide substrate and encapsulated (PI_encaps_/MoS_2_/PI_subs_) are shown in Figure 2a. The transfer matrix method (TMM) was used to model the optical absorption of the cell and the absorption of its individual layers.^44^ The absorption spectra between 400 and 800 nm wavelength are plotted to include the absorption in the visible range below the band gap ∼1.88 eV, clearly showing the modeled A, B, and C peaks of monolayer MoS_2_ at 652, 606, and 419 nm, respectively. The *n* and *k* data reported by Islam et al. were used to simulate the MoS_2_ monolayer absorption,^45^ while the CP1 polyimide n and k data were obtained from NeXolve.

### 2.3 Synthesis of MoS_2_ and PV Device Fabrication

MoS_2_ films were synthesized by chemical vapor deposition (CVD) in an MTI OTF-1200X-II dual zone split tube furnace with a third low temperature zone heated by a Grainger SLR series silicone heating blanket. ACS reagent ≥99.5% molybdenum(VI) oxide (MoO_3_) powder and 99.98% trace metal basis sulfur (S) powder precursors were used. More growth details are discussed in previous work.^45^ To fabricate the 2D solar cells, the contacts were patterned using a RAITH VOYAGER 100 electron beam lithography (EBL) tool, and the Ti and Pt contacts were both deposited using an Angstrom Engineering Nexdep electron beam evaporation tool. A surface energy-assisted film transfer process was used to transfer the monolayer MoS_2_ film from an Al_2_O_3_ substrate onto the contacts on the polyimide substrate. The complete steps are shown schematically in Figure S1 (Supporting Information).

### 2.4 Solar Cell Absorption and JV Characterization

The absorption measurements were obtained by using a PerkinElmer LAMBDA 750 UV/vis/NIR spectrophotometer equipped with an integrating sphere. The solar cell’s current–voltage characteristics are measured by using a Keithley 2450 sourcemeter. A TS-Space Systems Unisim five-zone solar simulator with tunable halogen and metal halide lamps and three tunable LEDs is used to simulate the 1 sun AM0 illumination conditions at room temperature.

### 2.5 Radiation Test

The radiation tests were conducted using a 5 MeV 150 kW electron accelerator at the NEO Beam Alliance-Mercy Plastics electron radiation facility. The electron beam was scanned over a test plate with the samples mounted, similar to a scanning electron microscope. Samples were grouped by exposure time for ease of planning. Once an exposure target was reached, the beam was paused and the relevant samples were removed from the test plate. This procedure was repeated until the full dose of 1 × 10^15^ electrons/cm^2^ was achieved for the third batch of samples, for a fluence on the three batches of 1.03 × 10^14^, 5.02 × 10^14^, and 1 × 10^15^ e/cm^2^. A thermocouple was mounted between the Faraday cup and test stage to monitor the temperature throughout testing. The maximum recorded temperature was roughly 40 °C as shown in Figure S10 (Supporting Information).

## 3 Results and Discussion

### 3.1 Optical Characterization and Modeling

The simulated monolayer MoS_2_ peak locations are blue-shifted relative to the peak locations of the experimentally measured MoS_2_ film. This is because the experimental absorption is measured for as-grown monolayer MoS_2_ on sapphire, while the *n* and *k* data used in the simulation are from variable angle spectroscopic ellipsometry (VASE) measurements of transferred monolayer MoS_2_ on an SiO_2_-on-Si substrate. Compressive strain relaxation of the MoS_2_ lattice is expected when the as-grown MoS_2_ is transferred;^46^ however, there is no MoS_2_ strain relaxation in the PI_encaps_/MoS_2_/PI_subs_ stack since the MoS_2_ lattice strain is maintained by the polyimide encapsulant, which is applied prior to the transfer step. The absorption measurements and simulations match closely both in spectra and relative absorption percentages for the three cases.

Most of the absorption in the PI_encaps_/MoS_2_/PI_subs_ stack results from the MoS_2_ monolayer layer absorption, where the 0.65 nm-thick absorber exhibits as high as ∼17% absorption at 430 nm and ∼9% total absorption in the 400–800 nm wavelength range. Early experimental evidence, as shown in Figure S2, indicates that stacking monolayers of MoS_2_ via surface energy-assisted film transfer results in additive absorption increases, rather than seeing a transition to indirect band gap MoS_2_. Based on this trend, we use TMM to calculate the absorption in a varying thickness stacked MoS_2_ absorber layer for a stand-alone MoS_2_ film, MoS_2_/PI_subs_ stack, and PI_encaps_/MoS_2_/PI_subs_ stack. We show that increasing the MoS_2_ thickness increases the absorption in the 400–800 nm wavelength range shown in Figure S3a (Supporting Information). The total absorption is shown in Figure 2b. In the stand-alone MoS_2_ absorber, a significant increase in absorption is seen from 0.65 to 3.25 nm (1 to 5 layers) with a less pronounced increase up to 11 nm. A slight decrease in absorption is shown from 11 to 28 nm thickness due to interference effects in the stack, followed by a steady increase up to 68 nm before leveling off at thicknesses above 94 nm. Similar absorption trends are shown for the MoS_2_/PI_subs_ and PI_encaps_/MoS_2_/PI_subs_ stacks, except that while the increase in the 1-to-5-layer thickness range is less dramatic, no decrease in absorption is shown in the PI_encaps_/MoS_2_/PI_subs_ stack as the thickness increases.

The short-circuit current density (*J*_SC_) from the MoS_2_ absorber in the PI_encaps_/MoS_2_/PI_subs_ stack, as a function of MoS_2_ thickness, was calculated by integrating the absorption with the AM0 1 sun extraterrestrial photon flux over the 280–800 nm range, assuming unity internal quantum efficiency (IQE). The *J*_SC_ increases with MoS_2_ thickness as shown in Figure 2b, showing that stacking monolayers will lead to a significant increase in absorption and *J*_SC_ of the MoS_2_ solar cells. A maximum *J*_SC_ of 17.71 mA/cm^2^ was calculated by using the 280–800 nm simulated absorption spectrum for a 100.1 nm (154 layer) MoS_2_ absorber.

The encapsulant absorption is minimal in the 400–800 nm range with ∼5% absorption at 400 nm, which quickly decreases to ∼0% at around 475 nm. The effect of the PI_encaps_ thickness on the absorption of an MoS_2_ monolayer in the 400–800 nm range is shown in Figure S3b (Supporting Information) and for a 100.1 nm (154 layers) stacked MoS_2_ absorber in Figure 2c. While the thin film interference from PI_encaps_ significantly changes the wavelength-dependent absorption of a monolayer MoS_2_, altering the thickness of the polyimide encapsulant up to 500 nm results in negligible changes in the total absorption for a 100.1 nm-thick MoS_2_ absorber as shown in Figures 2c and S3c (Supporting Information), suggesting that applying a polyimide coating for protective purposes should have no significant negative impact on the cell’s performance.

From the calculated *J*_SC_ for the PI_encaps_/MoS_2_/PI_subs_ stack, along with using 0.92 V *V*_OC_ and 60.4% fill factor (FF) consistent with the projections in a previous study for a 0.65 nm-thick, 1.85 eV-band gap MoS_2_ film,^18^ the estimated specific power as a function of MoS_2_ thickness was calculated for a solar cell including the polyimide substrate and encapsulant. Additional modeling parameters are included in the model section of the Supporting Information. This PI_encaps_/MoS_2_/PI_subs_ stack represents a fully encapsulated single cell unit, scalable to a full module with other units in series or parallel with interconnects. Model projections indicate that for a 7.1 nm-thick MoS_2_ absorber, ∼5 kW/kg can be attained, which is comparable to existing Si and GaAs flexible PV cells (which are not fully encapsulated into modules)^47−49^ and much higher than the 0.1 kW/kg specific power for ultrathin flexible Si modules.^50^ Specific power of our MoS_2_ flexible thin-film solar cell, which does include the encapsulating layers associated with full module construction, approaches 12.97 kW/kg at 100 nm thickness. These results show that the 2D TMDC PV has the potential to be the leading candidate for high-specific power applications such as those in space.

### 3.2 Electrical Performance

**Figure 3:**
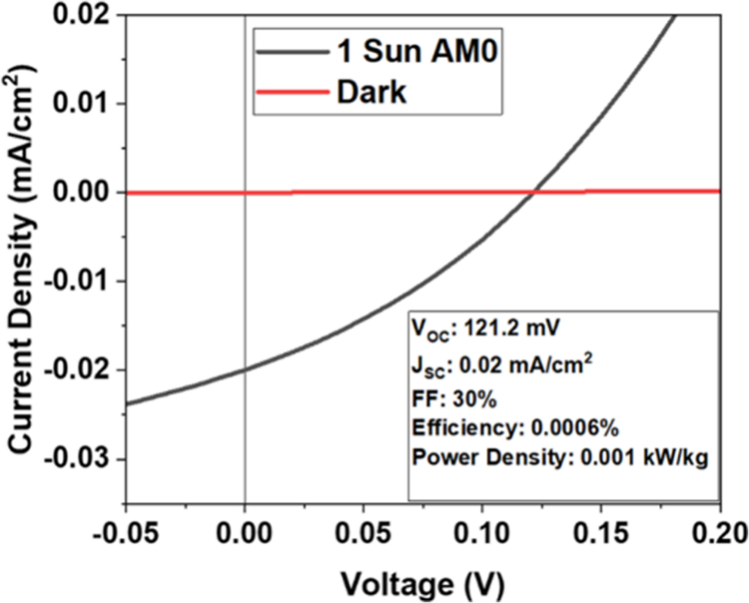
JV performance of a 0.0015 cm^2^-area monolayer MoS_2_ lateral Schottky-junction solar cell encapsulated in flexible polyimide under the dark and 1 sun equivalent AM0 illumination.

To characterize these flexible 2D MoS_2_ solar cells, 0.25 cm^2^ (as shown in Figure 1b)- and 0.0015 cm^2^-active area cells, sandwiched between polyimide layers, were fabricated as previously described, and the current density–voltage (JV) characteristics under AM0 1 sun illumination were obtained. The JV measurement for a device is shown in Figure 3, showing a *V*_OC_ of 0.121 V, *J*_SC_ of ∼0.2 mA/cm^2^, 30% FF, 0.0006% power conversion efficiency, and a specific power of 0.001 kW/kg. The average *V*_OC_, *J*_SC_, FF, efficiency, and specific power of the cells are also shown in Figure S4 (Supporting Information). A maximum *V*_OC_ of 0.180 V, *J*_SC_ of 0.02 mA/cm^2^, 31% FF, 0.0006% efficiency, and a specific power of 0.001 kW/kg were obtained for the solar cells. The low efficiency compared to the model can be attributed to higher recombination rates and lower carrier mobility in the films due to defects such as grain boundaries, contact Fermi-level pinning resulting in a lower Schottky barrier at the MoS_2_–Pt interface, and the small *J*_SC_ resulting from the low absorber thickness of 0.65 nm (monolayer MoS_2_). However, higher efficiency is expected for thicker active layers, and specific power is expected to increase by at least 2 orders of magnitude with a 100 nm-thick absorber layer made from stacking monolayer MoS_2_, as discussed previously (Figure 2d).

The current film transfer process involves manually transferring the films from the donor to the receiver substrate, which induces film damage and reduces device interface uniformity; for example, small air pockets form among the polyimide encapsulant, MoS_2_ film, contacts, and polyimide substrate, reducing the total MoS_2_ to contact coverage over the entire cell area, even after annealing. A more efficient and scalable transfer process is currently being explored to increase the absorber thickness to 100 nm or more, guided by our previously reported optical model to achieve efficiency acceptable for space PV. This in-progress work is beyond the scope of what is reported here. Nevertheless, the key metric that is being compared in this work is the full PV stack specific power, which is another critical metric for space energy systems.

Fermi-level pinning is also known to degrade *V*_oc_ in these devices relative to model expectations, although recent work shows pathways to reduce this effect.^51,52^ Finally, vertical transport devices provide a clear pathway to increase the internal quantum efficiency and reduce series resistance in these devices.^53,54^

### 3.3 Bending Test

**Figure 4:**
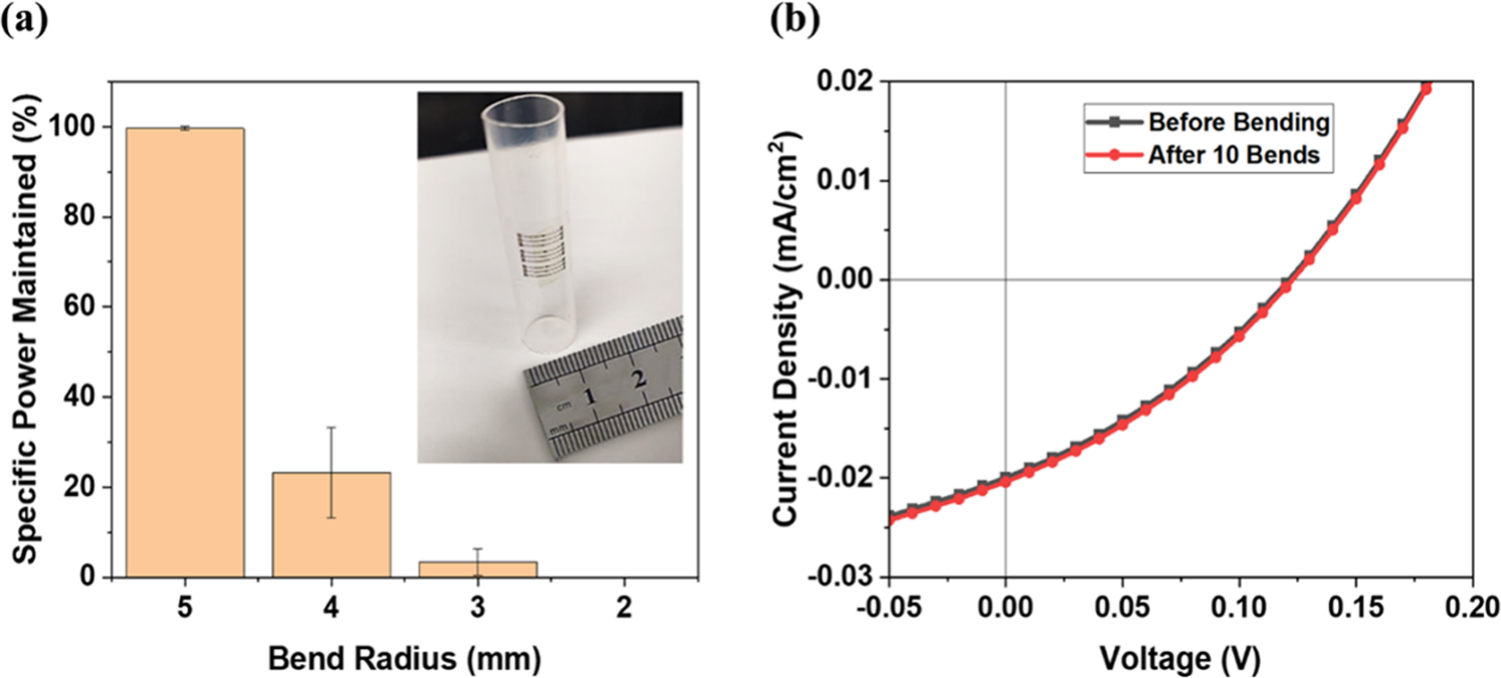
(a) Average specific power maintained (relative to prebend performance) vs bending radius of the flexible 2D MoS_2_ solar cells on polyimide substrates, with the inset showing the cells bent at 5 mm bend radius. (b) JV performance of a 0.0015 cm^2^ monolayer MoS_2_ solar cell under AM0 1 sun illumination before and after 10 bends at 5 mm bending radius.

An attractive property of 2D materials is their flexibility and the ease with which they can change substrates. For 2D PV in particular, this property can be leveraged for efficient storage and deployment, which makes 2D material-based PV desirable for space and other remote applications. To explore the robustness of our flexible cells and their ability to undergo a successful retraction and deployment process, the stack was wrapped around a tube of 5 mm bend radius 10 times, as seen in the inset of Figure 4a, and subsequently bent once at 4, 3, and 2 mm radius. The JV characteristics of the solar cells were measured after each bend. Due to the thin form factor of the stack, when the stack is bent, the stress on the stack can be approximated by the following equation
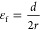
where *d* is the substrate thickness and *r* is the radius of curvature.^55^ At 5 mm bend radius, the ∼4.6 μm stack experienced 0.046% stress. A negligible reduction in the average specific power is observed, compared to the specific power before bending, across all of the solar cells when subjecting the devices to 5 mm bend radius. Furthermore, the *V*_OC_ of ∼0.122 V, *J*_SC_ of ∼0.21 mA/cm^2^, 31% FF, 0.0006% efficiency, and a specific power of 0.0011 kW/kg were consistent after 10 bending cycles as shown in the JV characteristic curves in Figure 4b. The JV characteristics after 10 consecutive bends of a 1 μm channel cell are shown in Table S1 (Supporting Information). However, further reducing the bend radius resulted in performance degradation across all of the samples. An average of 77% specific power reduction was measured for 4 mm bend radius, and a 100% reduction was seen for 2 mm bend radius, as shown in Figure 4a. Similar observations were made for the *V*_OC_, *J*_SC_, FF, efficiency, and the number of working cells as shown in Figure S5 (Supporting Information). The degradation of performance after bending to a smaller radius is explained by the formation of cracks in the contacts near the finger-busbar junction, resulting in open circuits in the cells. An image of cracks in the Ti contacts is shown in Figure S6 (Supporting Information); this failure mode is expected due to the sudden change in dimensions at this junction during bending. The test shows that the cell’s ability to maintain the same performance after bending is not currently limited by the 2D material’s resistance to bending but rather to that of the metal contacts, the design of which may be modified to improve bending performance. The results highlight the consistency in the performance of the 2D solar cells over multiple bends at 5 mm bend radius, which is desirable for array stowage at launch and deployment in space.

### 3.4 Radiation Testing

**Figure 5:**
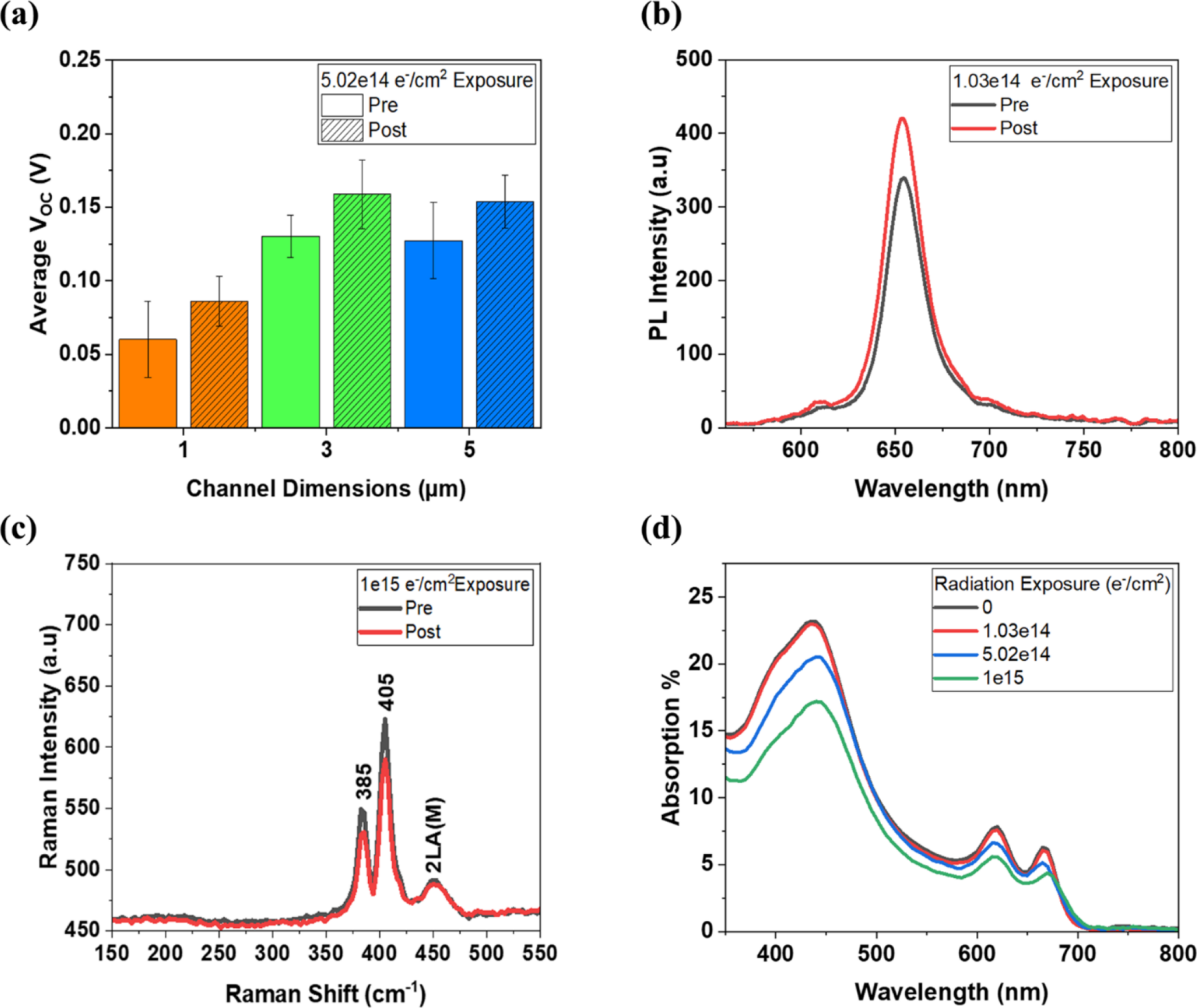
(a) Average *V*_OC_ before and after 5.02 × 10^14^ e^–^/cm^2^ of 1 MeV electron radiation exposure for the 2D MoS_2_ solar cells at different channel dimensions. (b) PL spectra of the as-grown monolayer MoS_2_ film before and after 1.03 × 10^14^ e^–^/cm^2^ of 1 MeV electron exposure. (c) Raman spectra of this film before and after 1 × 10^15^ e^–^/cm^2^ of 1 MeV electron exposure. (d) Optical absorption of this film before and after 1.03 × 10^14^, 5.02 × 10^14^, and 1 × 10^15^ e^–^/cm^2^ of 1 MeV electron exposure.

As mentioned previously, 2D TMDC materials are known for their exceptional radiation tolerance, and here, we validate the performance of these devices under radiation levels typical of low-Earth orbit conditions. Figure 5a–d shows the comparison of the solar cell’s average *V*_OC_, PL, Raman spectrum, and optical transmission spectrum before and after exposure to 1 MeV electrons at the NEO Beam Alliance Inc. electron accelerator facility. This facility’s space radiation testing capability was characterized and validated by the Monte Carlo analysis method for predicting damage from ion penetration in materials, meeting the AIAA standard S-111 qualification and quality requirements for space solar cells.^56−58^ The solar cells and as-grown MoS_2_ films on Al_2_O_3_ substrates without encapsulation were exposed to fluence levels of 1.03 × 10^14^, 5.02 × 10^14^, and 1 × 10^15^ e^–^/cm^2^. The first of these exposure doses is equivalent to 150 years in low Earth orbit (LEO), while the latter dose is equivalent to 15 years in the geostationary orbit (GEO), both using the displacement damage dose (DDD) method for space solar cell degradation analysis developed for GaAs and Si cells and devices.^56,59−61^ The average *V*_OC_ of the solar cells increased after exposure to all fluence levels, as shown in Figures 5a and S7d–f (Supporting Information). A similar trend is observed in the FF, with the exception of the 1 μm smallest channel cells (Figure S7g–i, Supporting Information). However, the increase in the *J*_SC_ was not consistent across all the devices (Figure S7a–c, Supporting Information). For example, the 3 μm channel cells post-5.02 × 10^14^ e^–^/cm^2^ exposure and the 5 μm channel cells post-1 × 10^15^ e^–^/cm^2^ exposure both had a decrease in average *J*_SC_. A possible explanation for decreased *J*_SC_ is the loss of the MoS_2_ film through slight film degradation or etching during exposure. Nevertheless, an average improvement in the efficiencies is observed for all exposures and channel lengths, as shown in Figure S7j–l (Supporting Information), with the largest increase in efficiency, by a factor of 2.2, occurring after the 1.03 × 10^14^ e^–^/cm^2^ exposure in Figure S7j (Supporting Information). The solar cell performance improvements are attributed to radiation-induced defect healing in the MoS_2_ film, similar to the observations made by Vogl et al., while studying the radiation resistance of WS_2_,^28^ or radiation-induced improvements in the PI_encaps_/MoS_2_/PI_subs_ and MoS_2_/metal contact interfaces. This defect healing is supported by the average ∼24% increase in PL shown in Figure 5b after exposing the as-grown MoS_2_ films to 1 MeV electrons with a fluence of 1.03 × 10^14^ e^–^/cm^2^. Figure S8a also shows an average increase in PL after 5.02 × 10^14^ e^–^/cm^2^ exposure of ∼15%, along with an average PL red shift of ∼2.28 nm that is seen. The red shift can be attributed to slight film delamination from the sapphire substrate during irradiance, resulting in strain relaxation. Slight PL reduction is observed after the 1 × 10^15^ e^–^/cm^2^ exposure, with more strain relaxation red-shifting the PL by ∼3.05 nm, corresponding to a 0.01 eV decrease in the band gap. The standard monolayer MoS_2_ A_1g_ to E_2g_^1^ Raman peaks, located at ∼405 and ∼385 cm^–1^, respectively, remained consistently after each fluence level, as shown in Figures 5c and S9a,b (Supporting Information). However, a decrease in the average Raman peak intensity ratio *I*(A_1g_)/*I*(E_2g_^1^) is observed, as expected for the relaxation of compressively strained 2D materials.^62,46^ The intensity ratio changes from ∼1.13 after the 1.03 × 10^14^ e^–^/cm^2^ exposure to ∼1.11 after the 1 × 10^15^ e^–^/cm^2^ exposure, which is indicative of some delamination and relaxation of the MoS_2_ film. Finally, a decrease in optical absorption and red shift of the absorption edge for the MoS_2_ films are observed with increasing radiation exposure, shown in Figure 5d. The red shift in the absorption edge correlates with the aforementioned compressive strain relaxation. The decreased absorption is likely due to the loss of some of the MoS_2_ material during the radiation process, which is likely offsetting the defect healing in the relatively lower PL increase for higher fluence levels seen in the PL data. This highlights the importance of encapsulation to mitigate the loss of active material after deployment of 2D PV in space. While interface investigations are beyond the scope of this work, we expect that both the material quality and the 2D semiconductor/contact interfaces have significant effects on the solar cell’s performance in the space environment. The beneficial effects of defect healing and interface treatment under radiation will eventually be overcome by material degradation from prolonged exposure, albeit at fluences associated with an exposure duration far beyond the expected lifetime of standard space PV.

### 3.5 Design and Techno-economic Analysis of 2D PV for Space Deployment

An ideal application for the 2D solar cell is to generate power for spacecrafts such as nanosatellites and CubeSats. The deployment of these miniature spacecrafts has grown significantly in recent years for several mission types, including interplanetary, low earth orbit (LEO), medium earth orbit (MEO), geostationary transfer orbit (GTO), geostationary orbit (GEO), and other missions. To date, over 2500 nanosatellites and 2300 CubeSats have been launched as of January 1, 2024, launch failures included.^63^ For an average mass of 1.5 kg per U (1000 cm^3^), the total estimated mass of launched CubeSats is greater than 7428 kg.^64^ Body-mounted solar panels can be used for low-power CubeSats, while deployable panels which are easily scalable, use lightweight backsheets for cell support, and feature compact opening mechanisms can be used to meet higher power demand CubeSats.^37^ Replacing conventional thin-film solar panels with ultrahigh-power density deployable 2D PV arrays can result in even higher power generation per mass while further reducing the payload volume at launch.

#### 3.5.1 CubeSat 2D PV Power System Design

**Figure 6:**
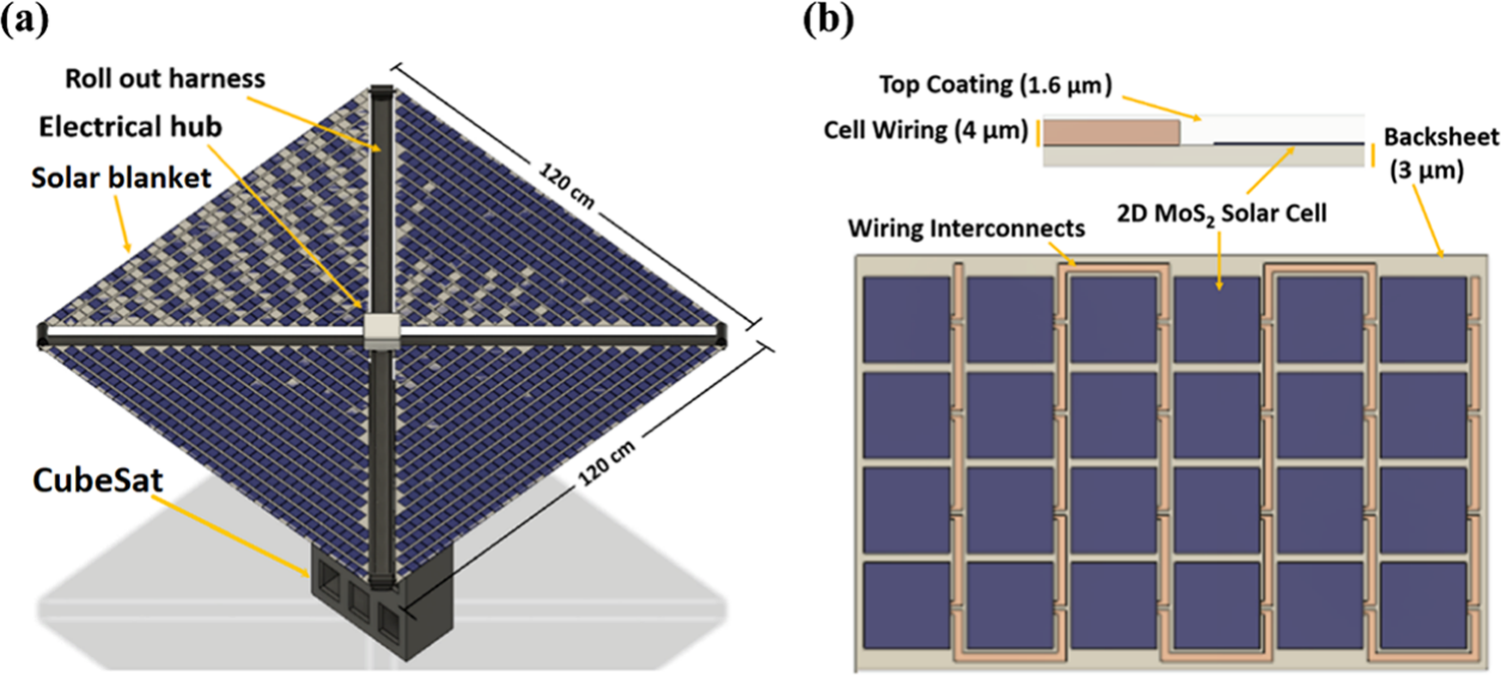
(a) Schematic of a 2D material-based solar power system deployed in space on a 6U CubeSat. (b) Schematic of the array cross-section and top view with cells and wiring on the polyimide backsheet.

A full-scale 2D material-based flexible 60 W solar power system compatible with a 6U CubeSat was designed as shown in Figure 6, based on the experimental subscale array prototype demonstrated in this work. The power generation system consists of four triangular cell arrays, composite polymer booms with stored strain energy for the array deployment (as implemented in the roll-out solar array, or ROSA),^65,66^ and a central electrical hub, all stowed in a 300 cm^3^ housing at launch.

Each triangular array is fabricated on the strong, tear-resistant colorless polyimide (CP1) substrate used in this work, which is similar to the material used for the NASA’s CP1TM solar sail produced by NeXolve.^43,67^ The design is also compatible with other array deployment mechanisms such as origami pattern deployment.^68,69^ The CubeSat with the solar arrays fully deployed is shown in Figure 6a.

#### 3.5.2 Array Deployment

Deployment of a 3 μm-thick PV array in an LEO presents a unique challenge and opportunity to an aerospace system integrator. The two main approaches to deployment for an ultrathin array are unfolding and unrolling. Using a compliant boom mechanism or a compliant array itself enables passive deployment and reduced system complexity.^68^ Folding patterns informed by origami researchers offer new and creative ways to stow systems, and 2D arrays can take unique advantage of these techniques due to their thickness and flexibility. The design shown in Figure 6a posits a compliant mechanism in the form of a high-strain composite boom that unrolls to unfold the array in a sail-like manner. This array architecture and deployment mechanism have been demonstrated at the technology readiness level (TRL) of 6 in practice,^70,71^ in a manner well-matched to a 2D material PV array. More mature deployment methods like the flexible composite boom we propose exist for traditional cells, most notably NASA’s ROSA, which has two successful deployments in an LEO on the International Space Station.

In our 6U CubeSat design, the 2D material-based solar cells include a 3 μm-thick polyimide backsheet with 4 μm thickness by 2 mm width gold cell interconnects fabricated on the same backsheet. The cell-to-cell interconnects are shown in Figure 6b. The front is coated with a 1.6 μm-thick transparent polyimide layer for cell encapsulation and structural support as shown in Figure 6b, which can be optimized as a flexible antireflection coating^72^ for increased absorption in the semiconducting layer. For this design, we use our previously modeled results for *V*_OC_ and FF, where each cell has a *V*_OC_ of 0.7 V, which is lower than the estimated 2D MoS_2_*V*_OC_ of 0.92 V to account for nonidealities, and a FF of 60.4%.^73^ The TMM-modeled *J*_SC_ of 16.61 mA/cm^2^ for a 78 nm-thick MoS_2_ absorber between polyimide layers is used here. Each cell is 10.24 cm^2^ in area and supplies 0.54 V and 12.91 mA/cm^2^ at the maximum power point. To achieve the target output voltage of >27 V, 51 cells are wired in series, with six parallel strings in each array blanket (four arrays in the total system). A total of 1224 cells are required to achieve the required 60 W output power by the end of life (EOL) (∼88 W at the beginning of life (BOL)), with a total solar power system area of 1.51 m^2^.

#### 3.5.3 Techno-economic Analysis

Assessing the practical feasibility of a new PV technology requires techno-economic analysis to compare against existing options. Two critical figures of merit for space-based PV are specific power (W/kg) and cost of power ($/W). Equation 1 is used to calculate the power cost, and eq 2 is used to calculate the specific power for the module.
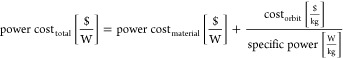
1
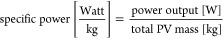
2Here, total PV mass includes the solar power-producing units but not the support structure or central electronics; for the 2D PV array, this includes the top coating, back sheet, cells, and interconnect wiring. Specific power (W/kg) informs the relative cost of launching a payload to the orbit and the power available to mass-constrained space missions. The cost of power ($/W) is derived from the manufacturing and launch costs of the PV power units and is an appropriate indicator of feasibility in space. The cost incurred launching mass to an LEO, $2720/kg for Falcon 9 as of 2018,^74^ dominates the cost of manufacturing the PV system, and so a more expensive system to manufacture can be economical if it has a higher specific power. This total power cost does not include integration expenses, which are challenging to model accurately and are borne by the spacecraft integrator and not the PV system manufacturer. Cost projections for commercialized 2D cells and arrays are modeled with a detailed bill of materials from supplier quotes and device design parameters with performance metrics based on the aforementioned modeled device with multiple layers of 2D MoS_2_. The benchmark space PV comparison used here is a SunPower monocrystalline Si PERC commercial panel.^39^ PERC cells are used by the space industry due to their relatively low cost of power ($/W) and competitive efficiency,^75^ making them a relevant benchmark for powering CubeSats in an LEO. All relevant parameters and calculated values for comparing the 2D PV array with a PERC panel are displayed in Tables S2–S6 (Supporting Information). In Table 1, a side-by-side technology comparison presents a 2D and silicon specific power of 6697.74 and 26.02 W/kg, respectively, when the masses of the metal contacts and interconnects are added. This specific power advantage of the thin-film design is also reflected in the total power cost (material plus launch) comparison, at $104.83 per watt for the PERC panel and $12.64 per watt for the 2D PV array. This specific power advantage demonstrates the practical potential for 2D nanomaterial-based PV to be used in mass-constrained environments like space. The advantage of 2D PV over conventional Si space PV is even greater when the support structure weight and cost are included. The results show that although 2D TMDC-based PV may have lower PCE, their significant advantage in specific power and cost to deploy in space, relative to the current most commonly deployed space PV technology, makes them a compelling option for further investigation.

**Table 1:** Comparison of the 2D PV Array with Si PERC Panel Deployment in Space

performance metrics	2D array	Si PERC panel
cost/area ($/m^2^)	$863.14	$21,238.94
weight/area (kg/m^2^)	0.0105	10.64
power/area (W/m^2^)	70.58	276.77
specific power (W/kg)	6697.74	26.02
cost/watt ($/W)	$12.64	$104.83

## 4 Conclusions

In summary, we present the design, modeling, and direct fabrication of large-area CVD-grown monolayer MoS_2_-based flexible PV devices on ultrathin, off-the-shelf 3 μm polyimide substrates with polyimide encapsulation, developed for deployment in space. The cell JV performance is characterized under a 1 sun AM0 simulated illumination source, and a maximum *V*_OC_ of 0.180 V, *J*_SC_ of 0.0198 mA/cm^2^, 31% FF, 0.0006% efficiency, and specific power of 0.001 kW/kg are achieved for the solar cells with a 0.65 nm-thick absorber. We explore the performance improvement of these PV devices with thicker MoS_2_ films. Model projections indicate that the polyimide encapsulant introduces negligible absorption in the 400–800 nm range, and with a 7.8 nm (12 stacked monolayers)-thick MoS_2_ absorber, ∼5 kW/kg can be attained, which is comparable to existing Si and GaAs flexible PV cells and exceeds existing modules. Furthermore, 12.97 kW/kg can be obtained for a 100 nm-thick MoS_2_ layer. The bending test reveals the ability of the flexible solar cells to maintain performance after multiple bending cycles to 5 mm bend radius, which is suitable for array stowage and deployment in space applications, and that both the metal contacts’ and 2D material’s resistance to bending is important in applications where the cells will be bent. Performance is shown to improve after radiation exposure to 1 MeV e^–^ fluence equivalent to 150 years in an LEO or 15 years in a GEO, which can be partially attributed to defect healing. Lastly, we present the design and techno-economic analysis of 2D PV for a 6U CubeSat. The results show that although 2D TMDC-based PV may have lower PCE, their specific power projects to be 2 orders of magnitude higher than a Si PERC panel, and the cost when deployed in space forecasts to be 1 order of magnitude less. This indicates that the flexible, large-area, high-specific power 2D material-based PV has great potential for space applications. Continued emphasis on improving 2D PV cell performance is critical to achieve large-scale adoption of 2D PV in space.

## Supporting Information Available

Schematic showing fabrication details for an ultrathin and flexible encapsulated MoS_2_ solar cell on NeXolve’s CP1 polyimide substrate; measured absorption spectra and computed absorption using the transfer matrix method for 1 to 4 stacked MoS_2_ monolayers; transfer matrix method (TMM)-simulated absorption spectra for the MoS_2_ layer; *J*_SC_, *V*_OC_, fill factor, and efficiency under 1 sun equivalent AM0 illumination; *J*_SC_, *V*_OC_, fill factor, and efficiency after bending; optical image showing a device after a 2 mm bend; *J*_SC_, *V*_OC_, fill factor, and efficiency before and after radiation exposure; photoluminescence spectra of the MoS_2_ film before and after radiation exposure; Raman spectra of the monolayer MoS_2_ film before and after radiation exposure; temperature profile of the testbed during radiation exposure; performance of devices before and after bending to 5 mm bend radius; and techno-economic analysis parameters (PDF)
