# Supplementary material for: High-Specific Power Flexible Photovoltaics from Large-Area MoS2 for Space Applications
Source: ACS Appl Energy Mater. 2025 Jan 2;8(1):87–98. doi: 10.1021/acsaem.4c01797 (PMC11733925; doi:10.1021/acsaem.4c01797)
Supplement: Supplementary file 1 — ae4c01797_si_001.pdf [file ae4c01797_si_001.pdf]

SUPPLEMENTARY INFORMATION

High-specific-power flexible photovoltaics from large-area MoS_2 for space applications

Timothy Ismael, Muhammad Aamir Abbas, Owen P. Harris, George B. Ingrish, Meghan E. Bush, Joshua M. Sasson, Jeremiah S. McNatt, and Matthew David Escarra**

T. Ismael, M. D. Escarra

Department of Physics and Engineering Physics, Tulane University,

New Orleans, LA 70118-5636, USA

E-mail: tismael@tulane.edu, escarra@tulane.edu

M. A. Abbas
Department of Physics and Engineering Physics, Tulane University,
New Orleans, LA 70118-5636, USA

O. P. Harris
Department of Physics and Engineering Physics, Tulane University,
New Orleans, LA 70118-5636, USA

G. B. Ingrish
Department of Physics and Engineering Physics, Tulane University,
New Orleans, LA 70118-5636, USA

J. M. Sasson
Department of Physics and Engineering Physics, Tulane University,
New Orleans, LA 70118-5636, USA

M. E. Bush
NASA Glenn Research Center,
Cleveland, OH 44135, USA

J. S. McNatt
NASA Glenn Research Center,
Cleveland, OH 44135, USA

To directly fabricate the two-dimensional (2D) photovoltaics (PV) on a 3 μm thick film of NeXolve's CP1™ Polyimide (PI), a polyvinyl alcohol (PVA) film was spin-coated at 500 rpm for 15 seconds, then 4000 rpm for 45 seconds onto an SiO_2/Si substrate from a 10% w/v PVA from Sigma-Aldrich in de-ionized water solution. The PVA was then baked on a hotplate at 180°C for 90 seconds. The PI substrate was placed on the coated PVA/SiO_2/Si and the wrinkles and air bubbles were smoothed out using the rubber tip of a stylus pen. The stack was then placed on a hotplate at 180°C for no more than 15 seconds to avoid over-curing the PVA, and again the rubber tip of a stylus pen was used to smooth out the film and remove gaps between the PI and PVA layers while on the hotplate. 10% w/v PVA film was then spin-coated again at 500 rpm for 15 seconds, then 4000 rpm for 45 seconds onto the PI/PVA/SiO_2/Si stack. The additional PVA layer serves as a protective layer for the PI from aggressive solvents during the resist coating and liftoff steps. 200 nm of 495 A4 PMMA resist, followed by 400 nm of 950 C4 PMMA resist, both from Kayaku Advanced Materials, were spin-coated on the PVA/PI/PVA/SiO_2/Si stack. The contacts were patterned using a RAITH VOYAGER 100 electron beam lithography (EBL) tool, and after development in 1:3 MIBK/IPA, an additional DI water development step at 30°C for 15 seconds was done to develop the exposed PVA. The 100 nm of contact metal (Pt) was deposited at a rate of 0.7 Å s^{-1} for 5 minutes, then 1 Å s^{-1} until 100 nm thickness was achieved, using an Angstrom Engineering Nexdep electron beam evaporation tool. Liftoff was done using ethanol at 70°C. The from spin coating the resist to liftoff were repeated as before to pattern the second contact metal (Ti) except this time reducing the resist curing temperature to 130°C for 90 seconds to avoid over-curing the PVA. After the contacts are patterned, The PI film with the patterned contacts is released in DI water at 95°C and then scooped up while floating in the DI water by a PMMA coated substrate. The as-grown MoS_2 on

sapphire substrate [1] is spin coated with a 1.6 µm thick PI film and transferred onto the contacts using a surface energy assisted transfer technique. [2] The stack was then placed on a hotplate at 120°C to bake off the DI water between the PI encapsulant and PI substrate layers and annealed for 1 hr. The PI film with encapsulated devices was then placed in DI water to release the stack from the substrate to have the free standing flexible 2D PV. The steps are shown schematically in **Figure S1**.

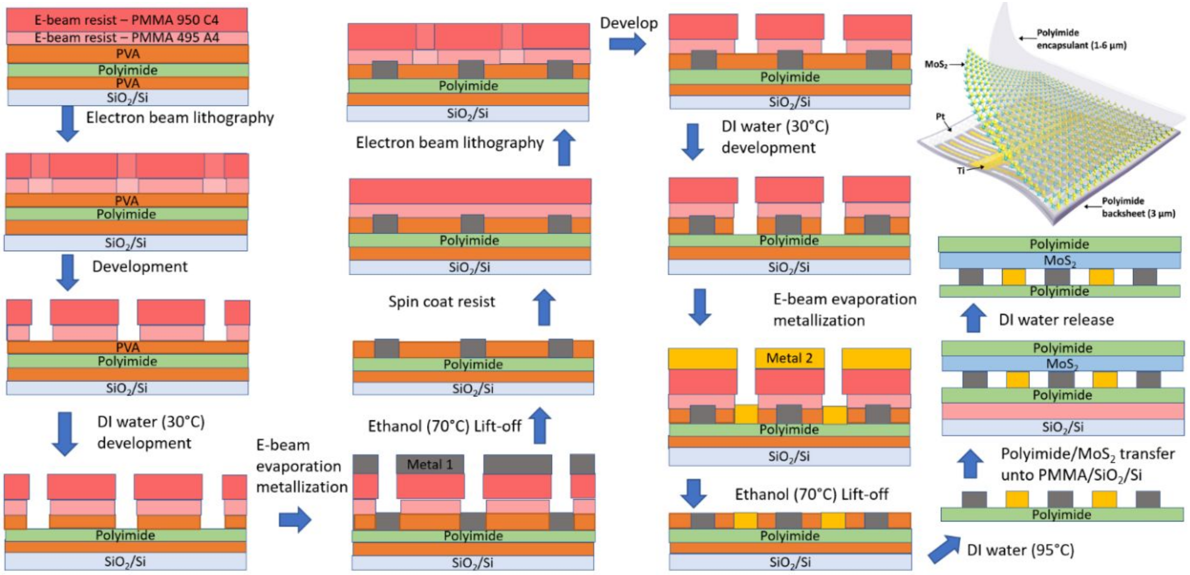

Figure S1. Schematic showing fabrication steps for an ultra-thin and flexible encapsulated MoS_2 solar cell on NeXolve's CP1™ Polyimide substrate.

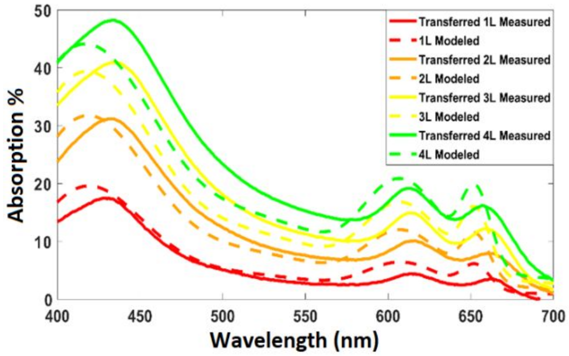

Figure S2. Measured absorption spectra and computed absorption using the transfer matrix method for 1 to 4 stacked MoS_2 monolayers. Absorption is shown to increase additively as a

result of layer-transfer-based stacking while maintaining the direct bandgap optical properties of MoS_2.

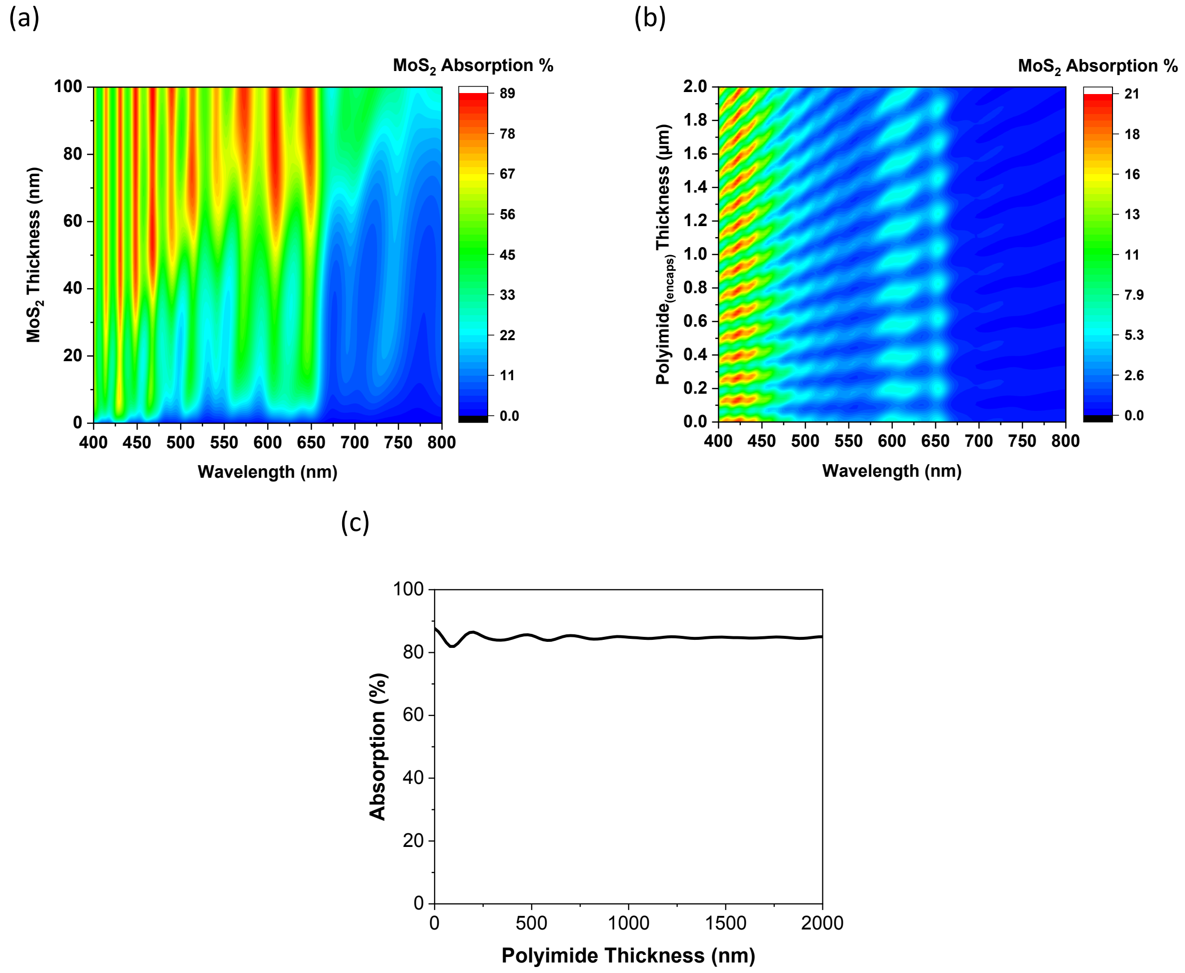

Figure S3 a) Transfer matrix method (TMM) simulated absorption spectra for the MoS_2 layer at different MoS_2 thicknesses in the $PI_{encaps}/MoS_2/PI_{subs}$ stack. b) Simulated absorption spectra in the 0.65 nm thick MoS_2 layer vs polyimide encapsulant thickness, c) total absorption in the 100 nm thick MoS_2 absorber vs. polyimide encapsulant thickness.

For the V_{OC} and FF calculations, The COMSOL Multiphysics Device model uses an MoS_2 film thickness of 0.65 nm, channel dimension of 1 μm, mobility of 1 $cm^2 V^{-1} s^{-1}$, and a donor concentration of 1×10^{18} cm^{-3}. [3] Additionally, a bandgap of 1.85 eV, electron affinity of 4.5 eV, relative permittivity of 3.5, electron effective density of states 2.66×10^{19} cm^{-3}, and hole effective density of states 2.86×10^{19} cm^{-3} were used for the MoS_2 layer. [4,5] Shockley-Read-Hall

(SRH) recombination is implemented to account for loss in the device, [6,7] The Ti and Pt work functions used were 4.33 and 5.65 eV respectively. For more information on our 2D material-based photovoltaic device modeling approach, please see our prior work.[8]

A total of 36 PV devices were fabricated with three channel lengths: 1, 3, and 5 μm with 0.15 mm^2 active area. **Figure S4** shows the average J_{SC}, V_{OC}, fill factor, and efficiency of the devices. A similar trend is observed where the device performance is dependent on the spacing between each Ti and Pt finger. [9] Decreasing the channel size increases the current under the same illumination because of the short diffusion length of the carriers in the MoS_2. For the 1 μm channels, more of the generated carriers reach the contacts before recombination, leading to a larger J_{SC}. The average performance of six devices before and after 10 bends at a 5 mm bend radius is shown in Table 1, indicating the robustness and flexibility of these modules.

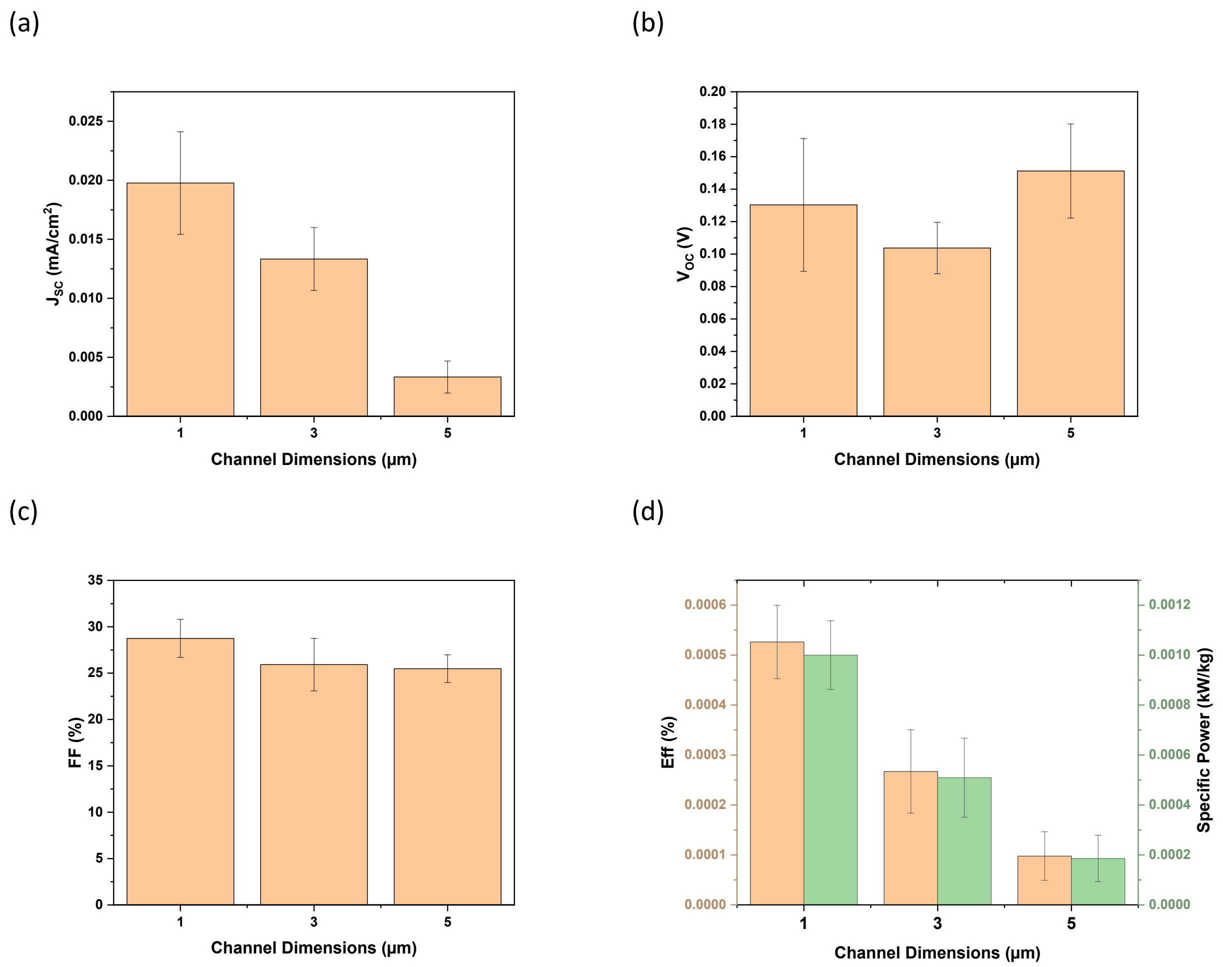

Figure S4. (a) J_{SC}, (b) V_{OC}, (c) fill factor and (d) efficiency and specific power vs channel length under 1 sun equivalent AM0 illumination.

Table S1. Average performance of 6 devices before and after bending to a 5 mm bend radius

	Before bending	Average after 10 bends	Std
**J_{SC} (mA/cm^2)**	0.0208	0.02093	4.21769E-4
**V_{OC} (V)**	0.1212	0.12171	0.00147
**FF %**	30.9	30.951	0.151
**Efficiency %**	5.77E-4	5.846E-4	2.12038E-5
**Power Density (kW/kg)**	0.0011	0.0011	4.07138E-5

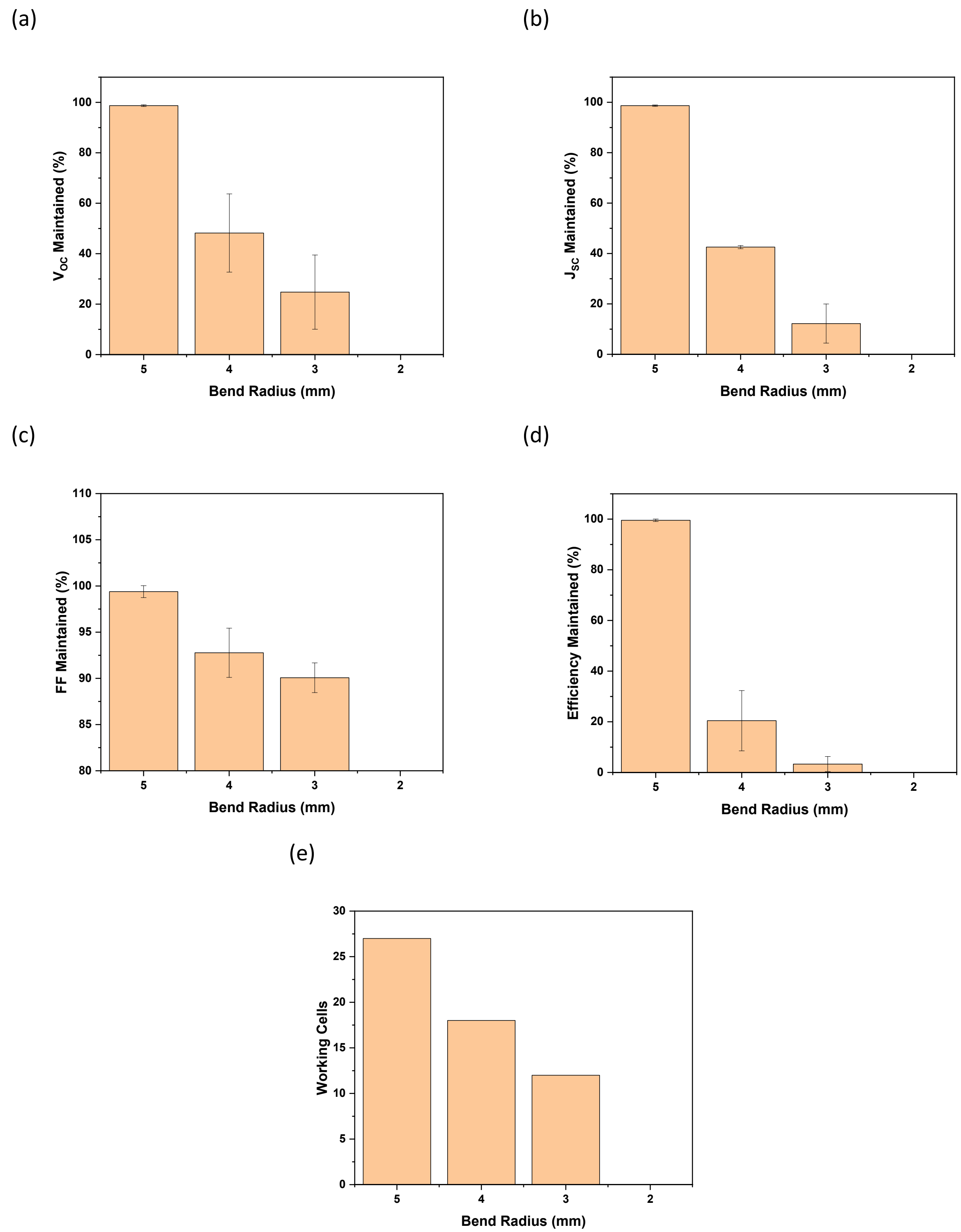

Figure S5. (a) V_{OC} (b) J_{SC}, (c) fill factor, (d) efficiency maintained (relative to pre-bend performance) and number of working cells vs bending radius for 27 devices under 1 sun equivalent AM0 illumination. 10 bends were applied at 5 mm bend radius, and 1 bend was applied at each of the subsequent smaller bend radii (performed in order of decreasing bend radius).

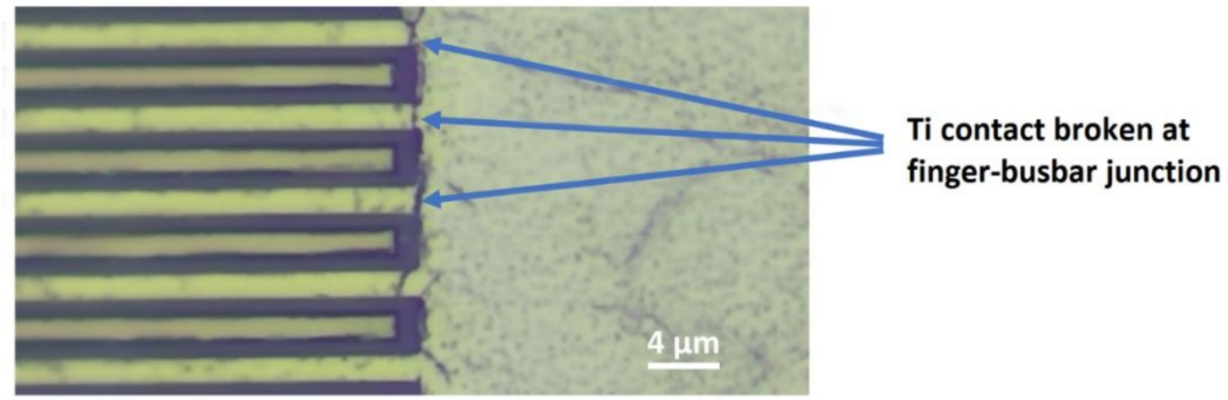

Figure S6. Optical image of broken Ti contact fingers at the finger-busbar junction where stress concentration is expected during bending. This image shows a device with 1 μm channel length after a 2 mm bend.

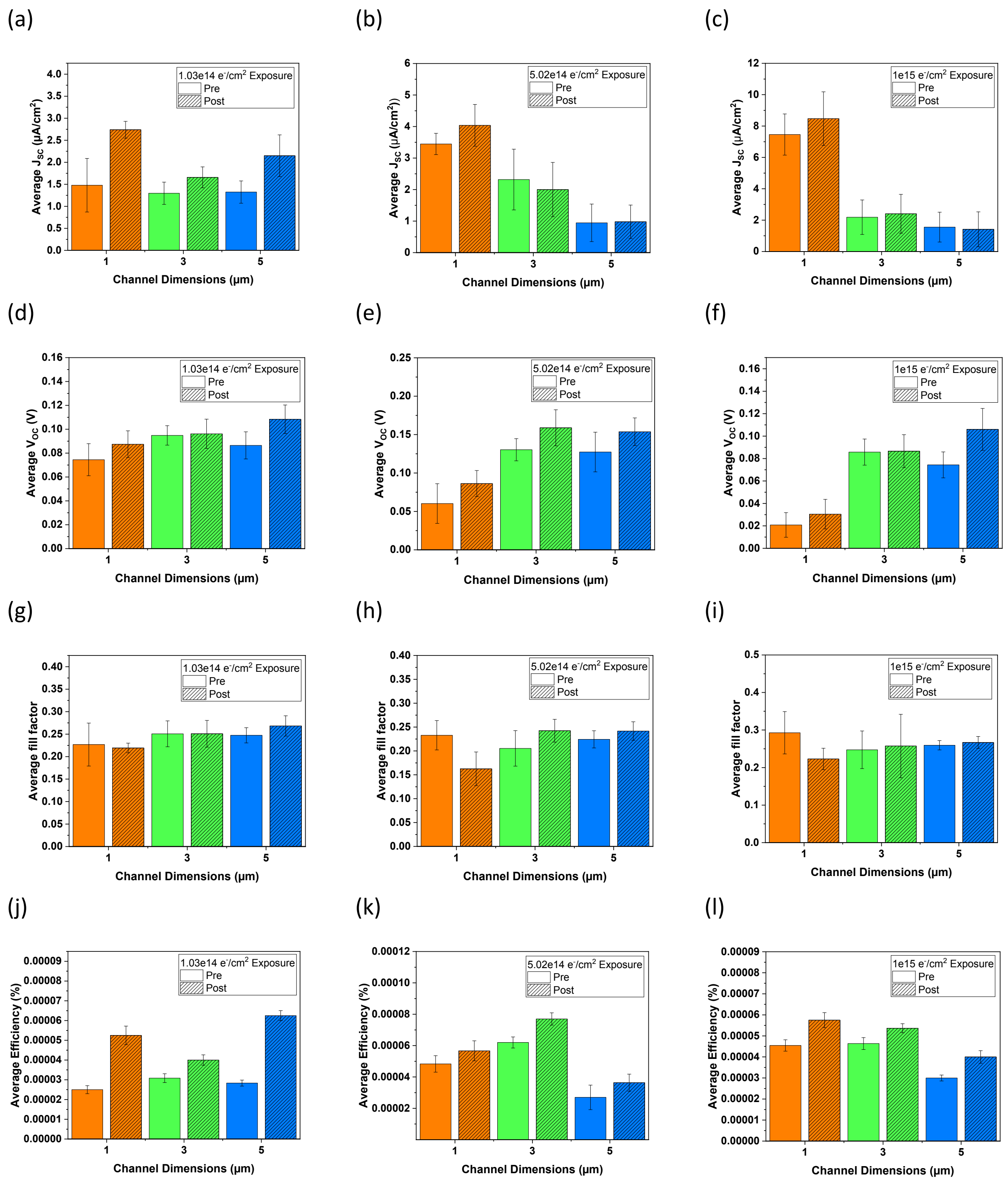

Figure S7. (a-c) J_{SC}, (d-f) V_{OC}, (g-i) fill factor, and (j-l) efficiency for 6 devices of each channel length (average and standard deviation given) under 1 sun equivalent AM0 illumination both before and after 1.03e14, 5.02e14 and 1e15 e^-/cm^2 of 1 MeV electron radiation exposure.

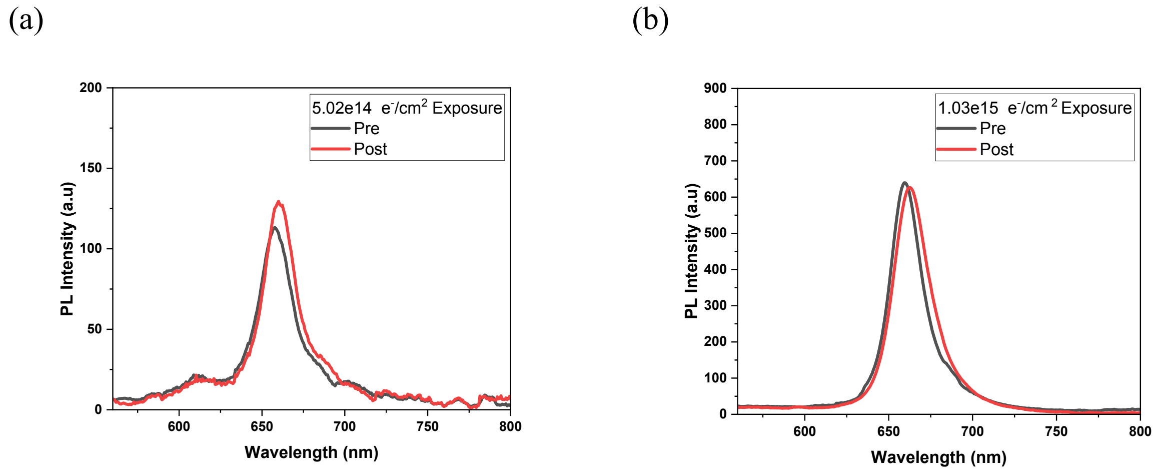

Figure S8. Photoluminescence spectra of the as-grown monolayer MoS_2 film before and after a) 5.02e14 and b) 1e15 e^{-}/cm^{2} of 1 MeV electron radiation exposure.

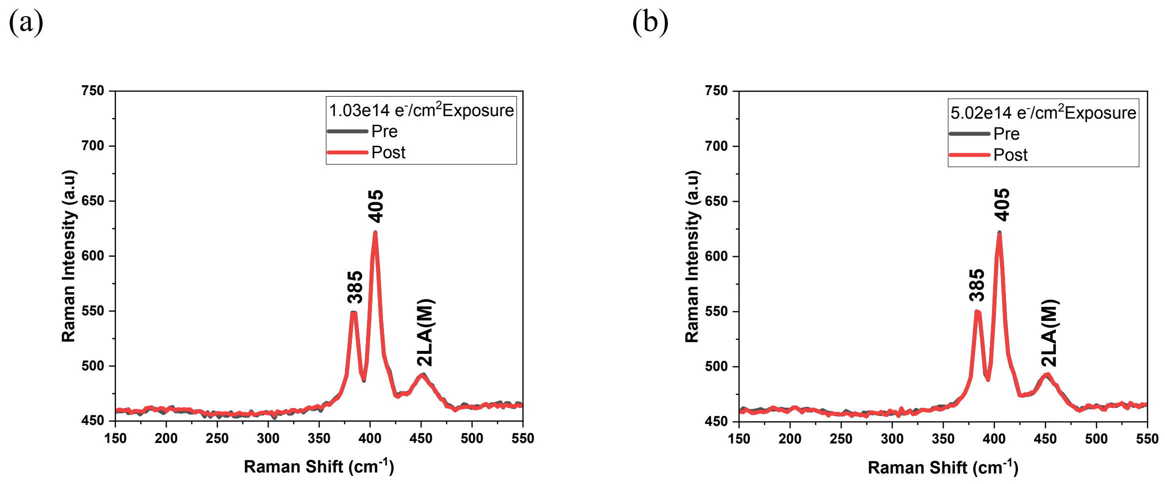

Figure S9. Raman spectra of the monolayer MoS_2 film before and after a) 1.03e14 and b) 5.02e14 e^{-}/cm^{2} of 1 MeV electron radiation exposure showing no difference in peak locations pre and post exposure.

Table S2. MASTER TECHNO-ECONOMIC ANALYSIS PARAMETERS

**Overall dimensions of each triangular array**		
**Base**	118.8	cm
**Height**	52.5	cm
**Cell Parameters**		
**Cell Length**	3.2	cm
**Cell Width**	3.2	cm
**Cell Thickness**	0.078	microns
**Cell Density**	5.06	g/cm^3
**Cell Fill Factor**	0.604	
**Cell V_{OC}**	0.7	Volts
**Cell Jsc**	16.614	mA/cm^2
**Cell Vmpp**	0.544	Volts
**Cell Jmpp**	12.911	mA/cm^2
**Cell Count/String**	51	
**Parallel Strings per Array**	6	
**Array Parameters**		
**Backsheet Thickness**	3	microns
**Backsheet Mass Density**	1.54	g/cm^3
**Top Coating Thickness**	1.6	microns
**Top Coating Mass Density**	1.54	g/cm^3
**Gold Wire density**	19.3	g/cm^3
**Wire Length**	700	cm
**Wire Width**	2	mm
**Wire Thickness**	3	microns
**Array Cell Height**	15	cells
**Array Cell Width**	36	cells
**Cell Vertical Spacing**	3	mm
**Cell Horizontal Spacing**	1	mm
**System Parameters**		
**Array Count**	4	
**Arrays in Series**	0	
**Arrays in Parallel**	4	

Table S3. Cell Computations

Cell Parameters		
**Area per Cell**	10.24	cm^2
**Volume per Cell**	7.99E-05	cm^3
**Total # of Cells**	1224	
**Cell Current**	132.22	mA
**Cell Voltage**	0.544	V
**Cell Power**	0.072	W
**Mass per cell**	0.87	mg
**Sulfur Precursor Mass**	50	mg
**MoO3 Precursor Mass**	1	mg
**Finger Volume**	3.19E-05	cm^3
**Busbar Volume**	4.02E-06	cm^3
**Platinum Density**	21450	mg/cm^3
**Titanium Density**	4506	mg/cm^3
**Platinum Mass**	3.86E-01	mg
**Titanium Mass**	8.10E-02	mg

Table S4. Array Computations

**Array Parameters**		
**Base**	118.8	cm
**Height**	52.5	cm
**Area**	3118.5	cm^2
**Side length**	79.276	cm
**Backsheet**		
**Area**	3118.5	cm^2
**Volume**	0.93555	cm^3
**Mass**	1.4407	g
**Top Coating**		
**Area**	3118.5	cm^2
**Volume**	0.49896	cm^3
**Mass**	0.76840	g
**Electrical**		
**Voltage**	27.75	V
**Current**	0.7933	A
**Power**	22.01	W
**Wire volume**	0.042	cm^3
**Wire mass**	0.8106	grams
**Total Array Mass**	3.286	grams

Table S5. Sunpower P-Series Commercial PERC

Part #	**SPR-P6-400-COM-XS** [10]		

**Specification**	**Value**	**Metric**	**Comment**
	**Dimensions**		
Weight	21	kg	
Length	1.092	m	
Width	1.808	m	
Area	1.974336	m^2	
	**AM1.5**		
Voc	36	V	
Isc	14.36	A	
Eff	20.30%		
Nom. Power	400	W	
Power cost (panel only)	$ 0.411	$/W	NREL[11]
	**AM0**		
Nom. Power	546.44	W	
Specific Power Density	26.021	W/kg	
Power cost (material only)	$ 0.30	$/W	AM0 adjusted
Launch Cost	$ 2,720.00	$/kg	Falcon 9 [12]
**Total Power Cost**	$ 104.83	$/W	
Material Cost	$ 164.40	$/module	
**Cost by area**	$ 21,238.94	$/m^2	

Table S6. 2D PV Bill of Materials

Bill of Materials				
	Value	Metric	Total Cost	Unit
**Total**				**/unit**
**Cell**			$ 0.7074	$/cell
**Molybdenum trioxide (MoO_3)**	0.000875	$/mg		
**Sulfur Powder**	0.00134	$/mg		
**Platinum**	0.03	$/mg		
**Titanium**	0.000138	$/mg		
**Array**			$ 52.71	$/array
**CP1 Backsheet**	0.0165	$/cm^2		
**Top Coating**	1.412	$/gram		
**Wiring**	0.206	$/cm		
**Launch**				
**Falcon 9** [12]	$ 2,720.00	kg	$ 8.94	launch cost/array
**Totals**				
**Array Cost to Orbit**	$ 278.11	$		
**Array Power Cost**	$ 12.64	$/W		

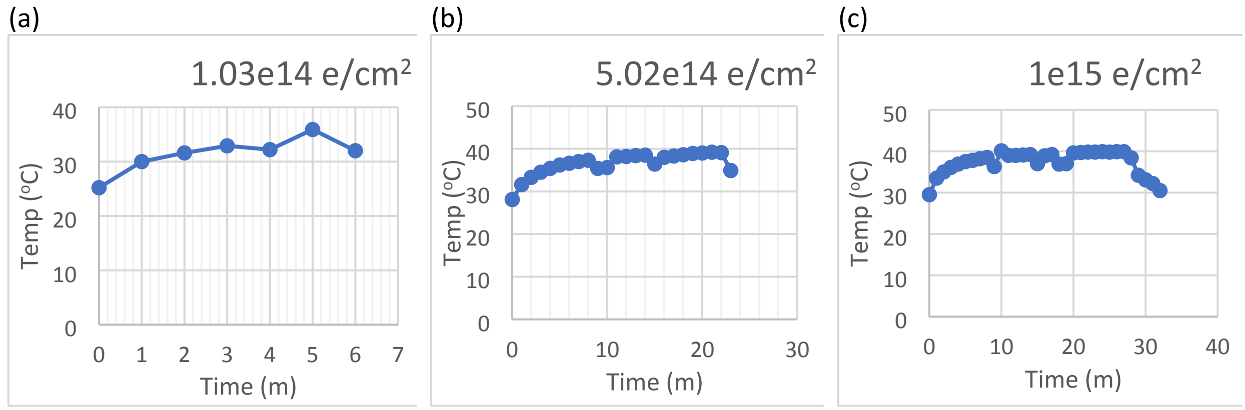

Figure S10. Temperature profile of the testbed during radiation exposure for a) 1.03e14, b) 5.02e14 and c) 1e15 e^-/cm^2 of 1MeV electron radiation exposure.

REFERENCES

(1) Ismael, T.; Grinalds, N. J.; Abbas, M. A.; Hill, N.; Luthy, C. E.; Escarra, M. D. Correlative Spatial Mapping of Optoelectronic Properties in Large Area 2D MoS2 Phototransistors. *Adv. Mater. Interfaces* **2023**, *2300455*. https://doi.org/10.1002/admi.202300455.

(2) Gurarslan, A.; Yu, Y.; Su, L.; Yu, Y.; Suarez, F.; Yao, S.; Zhu, Y. Surface-Energy-Assisted Perfect Transfer of Centimeter-Scale Films onto Arbitrary Substrates. **2014**, No. 11, 11522–11528. https://doi.org/10.1021/nn5057673.

(3) Dagan, R.; Vaknin, Y.; Henning, A.; Shang, J. Y.; Lauhon, L. J.; Rosenwaks, Y. Two-Dimensional Charge Carrier Distribution in MoS2 Monolayer and Multilayers. *Appl. Phys. Lett.* **2019**, *114* (10). https://doi.org/10.1063/1.5078711.

(4) Kumar, A.; Ahluwalia, P. K. Tunable Dielectric Response of Transition Metals Dichalcogenides MX 2 (M=Mo, W; X=S, Se, Te): Effect of Quantum Confinement. *Phys. B Condens. Matter* **2012**, *407* (24), 4627–4634. https://doi.org/10.1016/j.physb.2012.08.034.

(5) Kadantsev, E. S.; Hawrylak, P. Electronic Structure of a Single MoS 2 Monolayer. *Solid State Commun.* **2012**, *152* (10), 909–913. https://doi.org/10.1016/j.ssc.2012.02.005.

(6) Chu, Z.; Wang, C. Y.; Quan, J.; Zhang, C.; Lei, C.; Han, A.; Ma, X.; Tang, H. L.; Abeysinghe, D.; Staab, M.; Zhang, X.; MacDonald, A. H.; Tung, V.; Li, X.; Shih, C. K.; Lai, K. Unveiling Defect-Mediated Carrier Dynamics in Monolayer Semiconductors by Spatiotemporal Microwave Imaging. *Proc. Natl. Acad. Sci. U. S. A.* **2020**, *117* (25), 13908–13913. https://doi.org/10.1073/pnas.2004106117.

(7) McVay, E.; Zubair, A.; Lin, Y.; Nourbakhsh, A.; Palacios, T. Impact of Al2O3Passivation on the Photovoltaic Performance of Vertical WSe2Schottky Junction Solar Cells. *ACS Appl. Mater. Interfaces* **2020**, *12* (52), 57987–57995. https://doi.org/10.1021/acsami.0c15573.

(8) Islam, K. M.; Ismael, T.; Luthy, C.; Kizilkaya, O.; Escarra, M. D. Large-Area, High-Specific Power Schottky-Junction Photovoltaics from CVD-Grown Monolayer MoS2. **2022**, No. Cvd. https://doi.org/10.1021/acsami.2c01650.

(9) Islam, K. M.; Ismael, T.; Luthy, C.; Kizilkaya, O.; Escarra, M. D. Large-Area, High-Specific-Power Schottky-Junction Photovoltaics from CVD-Grown Monolayer MoS2. *ACS Appl. Mater. Interfaces* **2022**, *14* (21), 24281–24289. https://doi.org/10.1021/acsami.2c01650.

(10) Maxeon Solar Technologies. *Performance 6 solar panel SPR-P6-400-COM-XS*. https://sunpower.maxeon.com/au/sites/default/files/2023-04/sp_mst_p6_com_xs_1092_1.8m_.6m_cable_ds_en_a4_evo2_548188.pdf.

(11) Ramasamy, V.; Feldman, D.; Desai, J.; Margolis, R. U.S. Solar Photovoltaic System and Energy Storage Cost Benchmark: Q1 2021. *Natl. Renew. Energy Lab.* **2021**, No. September, 1–120.

(12) Jones, H. W. The Recent Large Reduction in Space Launch Cost. *48th Int. Conf. Environ. Syst.* **2018**, No. July 2018, 81.
